# Automatic Detection and Modeling of Underground Pipes Using a Portable 3D LiDAR System

**DOI:** 10.3390/s19245345

**Published:** 2019-12-04

**Authors:** Ahmad K. Aijazi, Laurent Malaterre, Laurent Trassoudaine, Thierry Chateau, Paul Checchin

**Affiliations:** Institut Pascal, UMR 6602, Université Clermont Auvergne, CNRS, SIGMA Clermont, F-63000 Clermont-Ferrand, France; laurent.malaterre@uca.fr (L.M.); thierry.chateau@uca.fr (T.C.)

**Keywords:** 3D point cloud, LiDAR, pipes, automatic detection, segmentation, portable 3D scanning system

## Abstract

Automatic and accurate mapping and modeling of underground infrastructure has become indispensable for several important tasks ranging from urban planning and construction to safety and hazard mitigation. However, this offers several technical and operational challenges. The aim of this work is to develop a portable automated mapping solution for the 3D mapping and modeling of underground pipe networks during renovation and installation work when the infrastructure is being laid down in open trenches. The system is used to scan the trench and then the 3D scans obtained from the system are registered together to form a 3D point cloud of the trench containing the pipe network using a modified global ICP (iterative closest point) method. In the 3D point cloud, pipe-like structures are segmented using fuzzy C-means clustering and then modeled using a nested MSAC (M-estimator SAmpling Consensus) algorithm. The proposed method is evaluated on real data pertaining to three different sites, containing several different types of pipes. We report an overall registration error of less than 7%, an overall segmentation accuracy of 85% and an overall modeling error of less than 5%. The evaluated results not only demonstrate the efficacy but also the suitability of the proposed solution.

## 1. Introduction

The underground space in urban areas has played an important role in urban development over recent years. Underground utilities are critical infrastructure for all modern cities. They carry drinking water, storm water, sewage, natural gas, electric power, telecommunications, etc. In most cities, this underground infrastructure reflects the growth and history of the city.

Mapping underground infrastructure has become indispensable for many important tasks pertaining to urban planning, safety and hazard mitigation, construction and maintenance, environmental impact, resource efficiency, etc. However, this offers several technical and operational challenges. The different infrastructure elements are usually placed at different depths resulting in lots of occlusions and overlap. The pipes and cables in the underground networks may well be conceptually simple, but they are narrow objects of different sizes, spanned over large geographical areas and made of multiple segments. For these very reasons the use of 3D technology from different scanning angles is more suited for such a task, as it allows a more accurate 3D mapping and modeling of the complex infrastructure. Several new regulations introduced recently in major cities impose a class-A geo-referencing/mapping (accuracy of 10cm on each axis) of all the sensitive underground networks in urban areas. This is essential, especially during construction and renovation tasks, where digging in the vicinity of safety-critical assets requires a high degree of vigilance from the construction workers and machine operators.

Today, for this task, surveyors are involved to conduct a manual survey at various stages when different underground infrastructures are laid. This is not only costly, but it lacks the necessary accuracy and it is also very time-consuming, considering the tight schedule of construction sites. To reduce the impact of these factors, automation is needed. The aim of this work is to develop a portable low cost automated mapping solution for the 3D mapping and modeling of underground networks during renovation and installation work, when the infrastructure is being laid down in open trenches.

## 2. Related Work

Underground mapping of pipe networks have been a topic of interest in the past several years now. Most of the pipe detection and mapping is done over the ground using ground penetrating radar (GPR) such as presented in [1,2,3]. However, dealing with open ditches with visible pipe networks is a different issue. Some works [4,5] have employed 2D camera images to detect and model these visible pipes in open ditches; however, these methods usually do not meet the accuracy requirements. The use of 3D light detection and ranging (LiDAR) sensors for mapping and modeling such visible pipe networks is quite recent. However, the problem of detecting and modeling pipes in industrial environment has received some attention in the scientific community in recent years. For instance, Rabbani et al. [6] presented a segmentation technique from LiDAR data employing a smoothness constraint based segmentation technique [6]. In this bottom-up approach, a *k*-nearest neighbors (*k*-NN) method is used to fit a plane to the neighbors to estimate the surface normal for each 3D point. The local surface curvature is then approximated using the residual of the plane fitting. Whereas a large residual indicates a curved surface, a small residual indicates a planar surface. A region growing method is then employed, in which points with the smallest residual are used as seed points, with neighboring points having residuals below a predefined threshold added to the current region based on the smoothness constraint. One of the shortcomings of this method is the computational complexity of the algorithm as *k*-NN needs to be computed for every point.

Liu et al. [7] presented a hierarchical structure detection and decomposition method to detect pipe-like objects in LIDAR data in oil refineries. In this method, the ground plane was first extracted using a Gauss map, and then the point cloud was projected onto the ground plane. In this method, the ground plane was first extracted using a Gauss map, and then the point cloud was projected onto the ground plane reducing the problem to finding circles in $R^{2}$, rather than finding pipes in $R^{3}$, using a random sample consensus (RANSAC) based method. In the first step, the detected pipes that were perpendicular to the ground plane were removed, and then the remaining points were projected onto different planes perpendicular to the estimated ground plane. A circle fitting technique was used to extract pipes parallel to the ground. The approach, which is computationally more efficient, resembles, in many ways, the joint cylinder segmentation and cylinder fitting approach. Also, similar to our proposed approach, it also considers pipes predominantly parallel to the ground plane.

In [8], an adaptive segmentation and extraction approach for planar and cylindrical features in terrestrial LIDAR data is presented. The method used eigenvectors and eigenvalues, computed from each point’s local neighborhood, to determine whether the points belong to planar or cylindrical surfaces. The local density variation and noise were taken into consideration for robustness. The directional and positional parameters of the cylindrical features were used to generate final segmentation results through clustering.

Masuda [9] proposed a method to recognize planes and cylinders from 3D industrial data. However, the method is semi-automatic as the region of interest has to be manually selected in advance. Matsunuma et al. [10] implemented a similar algorithm but it requires additional information such as brightness/intensity which is not always available. In [11], a skeletonization algorithm is used to classify scanned 3D point clouds into several groups, each corresponding to a single skeleton. Although the proposed method effectively extracts featured structures and also identifies connection relationships in the piping network, it somewhat struggles to find straight portions of the pipe in the point cloud. The authors of [12] used the skeleton extraction algorithm with Laplacian smoothing to determine the central axis of the pipe. This axis was then divided to estimate the curved region and the T shape of the cylinder. A multiple cylinder detection method that used a coarse-to-fine approach and a clustering algorithm was presented in [13]. As this method employed a 5D Hough transform to estimate the cylinder shape, it was computationally inefficient and so was generally computed separately in 2D and 3D. In [7], the data were first extracted and removed vertically and horizontally from the ground, and then the Hough transform was applied to project a plane on the normal vector followed by a cylinder shape estimation through circle fitting [14,15]. Huang and You [16] proposed a method to classify 3D point clouds using fast point feature histogram (FPFH) and support vector machine (SVM) and then segmented it using a classical flood-fill algorithm. In this, segmented data matching was done using RANSAC. However, the proposed method worked well mainly for relatively complex shapes having unique feature points. Similarly, in [17] primitive models were extracted in point clouds using FPFH, and Adaboost with library models was used to classify objects. The objects were merged and adjusted based on directionality and the start and end positions. Qiu et al. [18] computed a plane from normals (estimated through RANSAC) on the Gaussian sphere. The radius and length of the cylinder were estimated by projecting the segmented points on the plane. In order to estimate the cylindrical parameters of pipes, the authors in [19] employed RANSAC fitting to compute the center and radius of the sphere assuming to be the radius of the cylindrical axis of the pipe. Principal component analysis (PCA) was then employed to find the straight and curved portions of the pipes, using a discriminant based on eigenvalues. Where the curved portion was matched by a Catmull-Rom spline [20], the linear region was taken as the sum of the vectors of the linear candidates.

In addition to LiDARs, some studies have also employed RGB-D cameras, however, to little success. Nahangi et al. [21] proposed a radius estimation algorithm for accurate geometrical pipe feature detection, and using a radius feature metric, evaluated the performance of certain range cameras. The results showed an average error ranging from 10% to 18% at close distance measurements. Proença and Gao [22] proposed a method for fast plane and cylinder extraction to improve visual odometry performance on scenes made of cylindrical surfaces. In order to improve the efficiency and cater for sensor noise, image cells were used instead of 3D points. However, this resulted in surfaces smaller than the patch size to be filtered out. The authors demonstrated, in [23], a technique for the detection and pose estimation of arbitrary cylindrical structures in RGB-D data using a robust soft-voting scheme based on the generalized Hough transform. The rejection of outliers, mainly arising from planar surfaces that contaminate the orientation voting space, was improved by incorporating the curvature information in the voting scheme. However, the results were only evaluated on synthetic data.

The surface obtained after feature extraction needs to be represented and reconstructed. One of the most effective method is using implicit functions for this task [24,25]. This helps to generate tightly bound 3D models from discrete data points [26], repair missing data points, fill holes, and filter data acquisition noise [27]. However, for larger and more complex point clouds, estimating an accurate implicit equation is difficult. In our work, we divide the point clouds of segmented pipe structures into further subsections to estimate the parameters of the implicit function more accurately, similar to [28], in which the 3D point cloud is first divided into patches, and then each patch is fitted by different implicit functions before the fitting results are combined [29]. This technique, not only results in low computational load and a small amount of data, but it also allows local smoothing [28].

Most of these works either used single scans or an already registered point cloud, minimizing the role of registration, and they also focused more on a structured industrial environment. To the best of our knowledge, no work has directly used terrestrial LiDAR data to detect, map, and model pipes in open ditches in the outdoor environment. The unstructured environment poses several other challenges such as sparsity of point cloud, limited access and visibility, undue clutter especially due to vegetation and dirt/rubble, unforeseen occlusions, indiscriminating surfaces, etc. In this work, we use 3D data to detect, map, and model underground pipe networks during renovation and installation work when the infrastructure is being laid down/examined in open trenches, using a portable data acquisition system (explained in Section 3.1). Different scans obtained from the system are first registered together using a modified global iterative closest point (ICP) method to form a complete 3D point cloud of the scene (Section 3.2). This 3D point cloud is then analyzed to extract the ditch region, and then, within the ditch, pipe-like structures are segmented, employing fuzzy C-means clustering (Section 3.3). Different parameters of the segmented pipes are estimated using a nested MSAC (M-estimator SAmpling Consensus) method and then used to model the pipe network (Section 3.4). The results are evaluated on real data obtained from different sites to show the efficacy and viability of the method (Section 4). After a thorough analysis of the proposed method and relevant discussion, the conclusion is presented in Section 5.

## 3. Materials and Methods

### 3.1. Portable 3D Data Acquisition System

**Figure 1:**
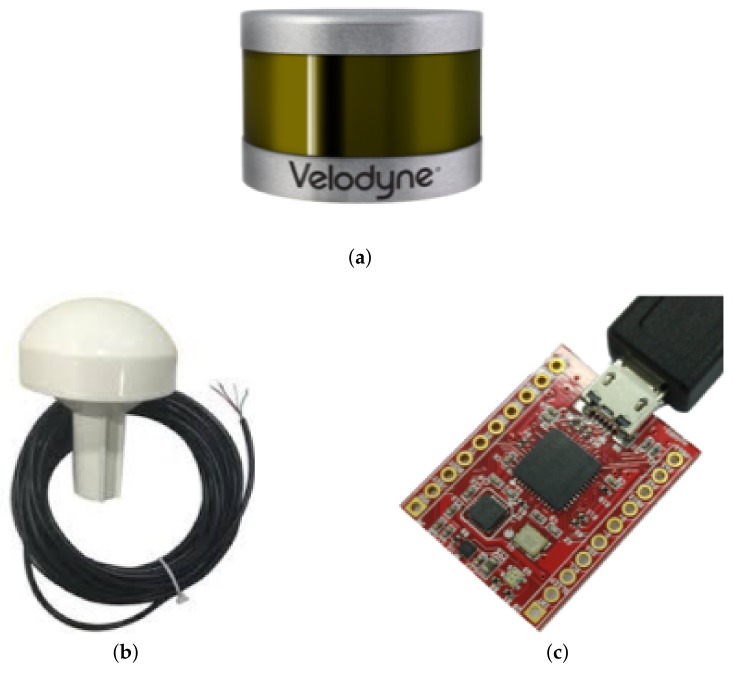
VLP-16 LiDAR (**a**), GPS receiver (**b**), and low cost attitude heading and reference system (AHRS) (**c**) used in the portable 3D data acquisition system.

The portable system used to collect data consists of a VLP-16 [30] laser scanner, a low cost AHRS (Attitude Heading and Reference System) [31], and a simple low cost Global Positioning System (GPS) receiver [32] as shown in Figure 1.

The VLP-16 is the third generation laser sensor produced by Velodyne. The salient features of the laser scanner are presented in [30]. Mounted on the same rigid assembly, the AHRS provides the rough orientation of the 3D scans, while the GPS provides its rough position, using rigid transformations of the later sensors in the VLP-16 frame of reference.

**Figure 2:**
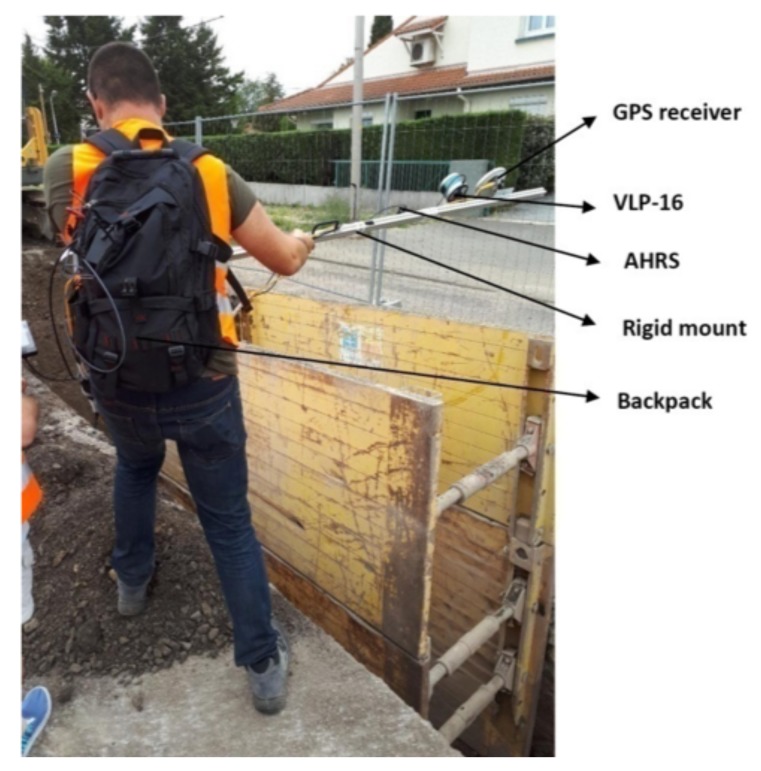
Scanning a ditch using the portable 3D data acquisition system.

Due to the limited vertical FOV (field of view) of the VLP-16 sensor, the user walks around the ditch extending the sensor assembly over it to effectively capture/scan the pipe network inside as shown in Figure 2.

The data is acquired and stored on the computer (laptop) which is placed in a backpack along with the portable battery. The total weight of the sensor assembly is about 2kg which makes it easy to carry and maneuver. The average total acquisition time for a ditch of about 5m in length is about 4min.

The rough orientation and position of the 3D scans help to provide an initial guess/alignment for multiple scan registration to form a single registered 3D point cloud, as explained in the next section.

### 3.2. Registration of 3D Scans

The rough alignment with the help of the AHRS and GPS data was not enough to form a coherent registered 3D point cloud. Thus, this initial information was used to finely register the acquired 3D scans to form a registered 3D point cloud using a modified global ICP technique as explained below.

**Figure 3:**
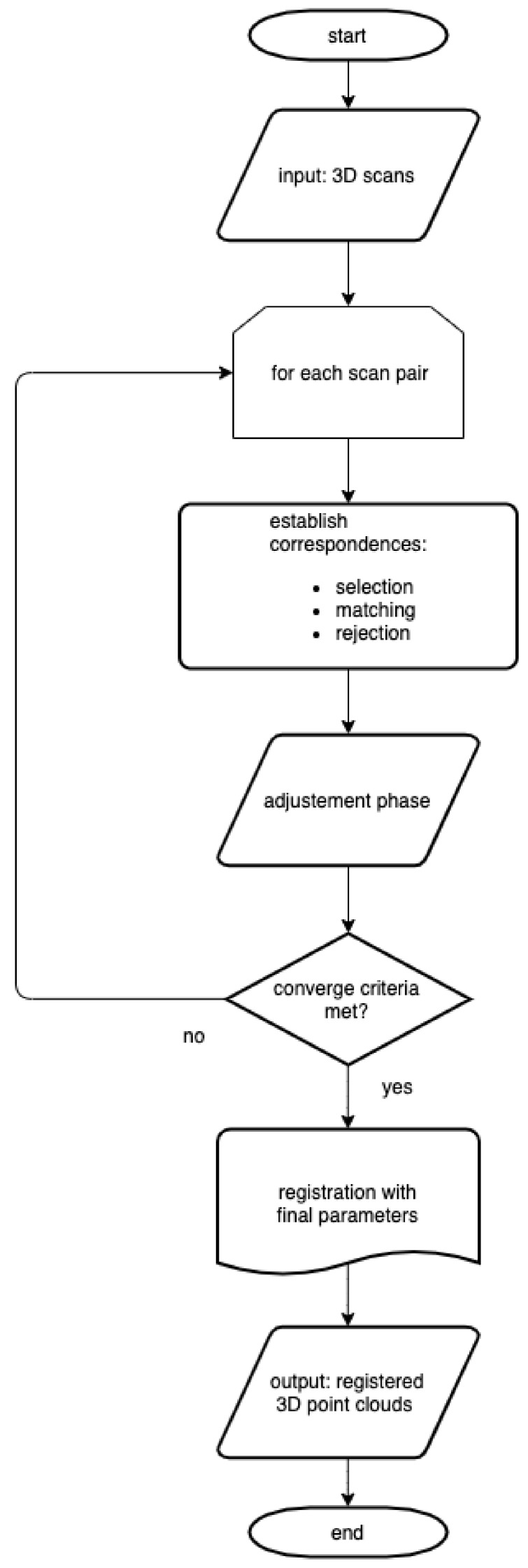
Summary of the proposed registration method.

In the global ICP the different 3D scans are finely registered using a type of bundle adjustment method as presented in [33]. The proposed method takes into account all the 3D scans at the same time and searches correspondences in the overlapping regions of different scans. Due to the fact that the LiDAR has a fast scan rate (10Hz), that the sensor assembly is moved quite slowly over the ditch (normal walking speed), and that the FOV of the sensor remains focused on the ditch during the whole scanning process, there is a large amount of overlap (overlapping regions) found in the different scans and hence several correspondences. The method is summarized in Figure 3.

It is shown in [34] that the registration results are strongly affected by the choice of correspondences. A single correspondence is defined by two points from overlapping regions along with their normal vectors (estimated from the neighboring points using a principal component analysis method [35]). As a point and its normal vector define a tangent plane, thus, a correspondence represents two homologous tangent planes in object space.

The correspondences are established for each pair of overlapping regions in three distinct steps: the Selection, Matching, and Rejection steps.

In the selection step, a subset of points is selected within the overlap region using maximum leverage sampling (MLS). This technique helps select those points, which are best suited for the estimation of the parameters. For this, the effect of each point on the parameter estimation, i.e., its leverage, is considered. The points with the maximum leverage, which correspond to the lowest redundancy, are selected. This strategy considers the coordinates and the normal vectors of the points.

The matching step establishes the correspondences. Each selected point from the selection step is paired to the nearest neighbor (the closest point) in the overlapping scans. The nearest neighbor search is efficiently realized using short for *k*-dimensional trees (*k*-d trees). As in the adjustment phase the point-to-plane distance is minimized for each correspondence, two associated points do not have to be identical in the object space, they only have to belong to the same (tangent) plane.

The rejection step aims to identify and subsequently reject unreliable or false correspondences. Based on our application and type of data, each correspondence is tested using the following criteria:

- Plane roughness of corresponding points: In order to minimize the point-to-plane distance, the reliability of both normal vectors must be ensured. This condition is not met if the scanned object cannot be appropriately modeled by a plane, e.g., in the case of vegetation.
- Angle between the normal vectors of corresponding points: To ensure that two corresponding points belong to the same plane, the angle α between the normal vectors of these points should not exceed an upper limit $\alpha_{\max}$. The value of $\alpha_{\max}=5{}^{\circ}$ is used in this work.
- Point-to-plane distance between corresponding points: Apart from a few false correspondences, the a priori point-to-plane distances $d_{1},d_{2},\ldots ,d_{n}$ are assumed to be normally distributed for each individual pair of overlapping scans. A robust estimator for the standard deviation of this contaminated set of correspondences is given by: $\sigma_{\mathrm{med}}=1.4826\times \mathrm{med}$, where med is the median of the absolute differences (with respect to the median) [36]. In this work, all correspondences with a point-to-plane distance outside the range of $\tilde{d}\pm 3\times \sigma_{\mathrm{med}}$ are rejected, where $\tilde{d}$ denotes the median of the point-to-plane distances of all correspondences that passed the first two criteria.

These criteria ensure that only the most reliable correspondences are retained. As is evident from these criteria, most of the correspondences belonging to structured parts of the environment are used whereas unreliable correspondences belonging to unstructured environment such as vegetation are rejected. Even if some false correspondences still remain after these rejection criteria, a robust adjustment method used helps in their detection and removal.

In the adjustment phase a robust least squares adjustment is performed to minimize the weighted sum of squared point-to-plane distances (which is defined as the perpendicular distance from one point to the tangent plane of the other point):

(1) $$\sum_{i=1}^{n}\left(w_{i}{d_{i}}^{2}\right)\to \;\mathrm{minimum}$$

where $w_{i}$ is the weight, and $d_{i}$ is the point-to-plane distance of the *i*-th correspondence. The signed point-to-plane distance $d_{i}$ is defined as the perpendicular distance from a point to a plane. It is conveniently expressed by the Hessian normal form:

(2) $$d_{i}={\left(p_{i}-q_{i}\right)}^{T}\cdot n_{i}$$

where $p_{i}$ and $p_{i}$ are the corresponding points of the *i*-th correspondence, and $n_{i}$ is the normal vector associated to the point $p_{i}$.

The weight of the correspondences $w_{i}$ is estimated iteratively (i.e., before each adjustment) from the point clouds itself or, more specifically, from the previously established correspondences. Assuming that the correspondence *i* belongs to the scan pair *k*, then its weight is determined by:

(3) $$w_{i}=\frac{1}{\sigma_{k}^{2}}$$

where $\sigma_{k}$ is the $\sigma_{\mathrm{med}}$ value of all (non-rejected) point-to-plane distances belonging to the scan pair *k*.

**Figure 4:**
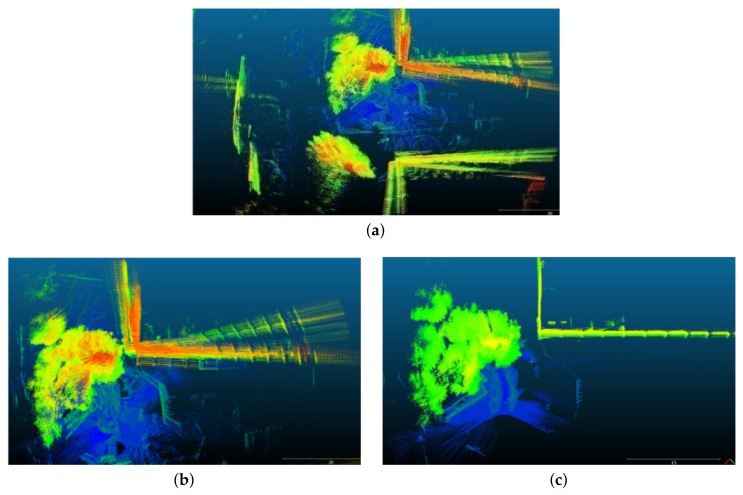
(**a**) The unregistered 3D point clouds of our Site-1 in the city of Grenoble, (**b**) a zoomed portion of the point cloud, (**c**) the registered 3D point cloud after the proposed registration process. Colors represent the elevation of the 3D points with blue representing the lowest and red the highest elevation.

**Figure 5:**
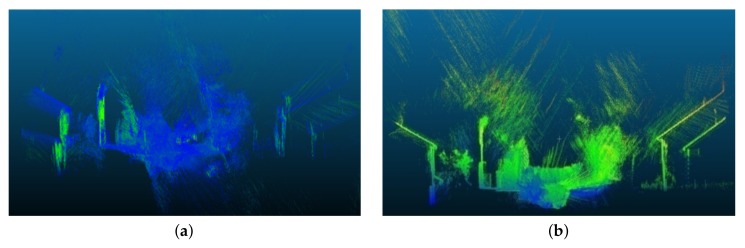
(**a**) The unregistered 3D point clouds of our Site-3 in the city of Pérignat-lès-Sarliève (**b**) the registered 3D point cloud after the proposed registration process. Colors represent the elevation of the 3D points with blue representing the lowest and red the highest elevation.

We find that although we are mainly concerned with 3D points belonging to the ditch, the ditch points are not fully used in the registration phase as many of these are rejected due to the strict rejection criteria. This is why the choice of a longer range sensor such as VLP-16 (range >100m) is useful for our application, as it allows scanning parts of more structured environments (for example, building facades, sign boards, and roads), which are predominantly used for registration (see Figure 4 and Figure 5 for some qualitative results).

### 3.3. Segmentation of 3D Point Clouds

Once the different scans are registered together to form a 3D point cloud, it is segmented to extract objects/points of interests. The segmentation process consist of two main steps:

#### 3.3.1. Semantic Filtering

**Figure 6:**
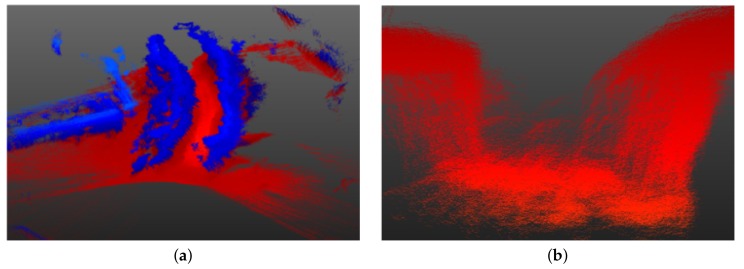
(**a**) The delimited zone around the ditch, (**b**) the extracted ditch points after analysis of height profile in the *X*–*Y* plane.

In order to extract pipes, we are only interested in the 3D points belonging to the ditch. So in order to extract the part of the point cloud belonging to the ditch, we apply certain semantic filters. The filters are based on distance, height, and profile. As we know that the scanning was done around the ditch itself, we only take into account the 3D points with horizontal and vertical distance of less than 5m. As the scanning range of the VLP-16 scanner used is >100 m, this pre-filtering helps us to remove large amount of unwanted 3D points from our analysis. This reduced 3D point cloud still contains some unwanted 3D points belonging to the ground/vegetation surrounding the ditch; hence, a profile based filtering is introduced to further narrow down the desired 3D points in the point cloud. Using a simple sweep scan method, as employed in [37], we analyze the height profile (*Z* direction) of the 3D point clouds with respect to the plane (*X*,*Y*) as shown in Figure 6. A crater usually represents the points belonging to the ditch. These 3D points are then extracted and used for further analyses.

#### 3.3.2. Fuzzy C-Means Clustering

Once the 3D points belonging to the ditch are extracted, they are segmented/clustered to form potential objects. In such a non-structured environment, the clustering/segmentation task, especially if only based on 3D information, can be quite difficult. This type of 3D data is widely hampered due to jump edges [38], feature occlusions [39], varying point resolution [40], close objects of large dimensions, and measurement noise [40], etc. For these reasons, a more robust method of modified fuzzy C-means clustering is employed in this work. Compared to other conventional methods, it is more suitable for this type of data. The originally conceived method [41] has been widely implemented (both in original and modified forms) in 2D image segmentation, especially for medical analysis applications [42,43]. Recently it has also been employed in the 3D domain with success [44,45].

Evolved from hard C-means (HCM) [46], fuzzy C-means (FCM) is a clustering method, in which each data point can belong to multiple clusters with varying degrees of membership. HCM allows only binary classification (0 or 1 according to distance) which makes it simple and fast, however, the precision can be lacking. Based on HCM, the proposed FCM method modifies the class membership function from {0,1} to [0,1]. As a result, each sample point has a specific membership degree relative to each of the class, and for each point, the sum of these membership degrees (one membership degree for every class) is 1.

Fuzzy clustering aims to minimize the following cost function as presented in [46]:

(4) $$J_{m}\left(U,V\right)=\sum_{j=1}^{n}\sum_{i=1}^{c}u_{ij}^{m}d_{ij}^{2}.$$

The sample space $X=\left\{x_{1},x_{2},\ldots ,x_{n}\right\}\subset R^{s}$, where *s* and *n* are the dimension and the number of samples respectively. *c* is the number of clustering classes, m>1 is the fuzzy factor, $d_{ij}=∥x_{j}-v_{i}∥$ is the distance between sample point $x_{j}$ and *i*-th cluster center $v_{i}$. $V={\left[v_{ij}\right]}_{c\times s}$ with $v_{i}\subset R^{s}$. $U={\left[u_{ij}\right]}_{c\times n}$ is the fuzzy membership matrix satisfying Equation (4) with $u_{ij}$ being the membership degree of $x_{j}$ belonging to the *i*-th class satisfying the following conditions:

(5) $$\left.\begin{array}{cc} \sum_{i=1}^{c}u_{ij}>1, & \;1\leq j\leq n\\ u_{ij}\geq 0, & \;1\leq i\leq c,1\leq j\leq n\\ \sum_{j=1}^{n}u_{ij}>0, & \;1\leq i\leq c \end{array}\right\}.$$

Thus, FCM can be considered as a constrained optimization problem with the following main steps [46]:

**Step** **I** **Initialization**
  - Cluster center $V^{\left(0\right)}$.
  - Iterative index k=0.
  - Maximum iteration number *K*.
  - Threshold ϵ>0.
**Step** **II** **Calculation of the membership degree matrix **U(k)
  - Given $\forall j,\;r,i\neq r,\;d_{ij}\left(k\right)>0$, then
    (6) $$u_{ij}=\frac{1}{\sum_{r=1}^{c}\left[{\left[d_{ij}\left(k\right)/d_{rj}\left(k\right)\right]}^{\frac{2}{m-1}}\right]}.$$
  - If there exist *j* and *r* values that make $d_{ij}\left(k\right)=0$, then
    (7) $$u_{rj}\left(k\right)=1,\;u_{ij}\left(k\right)=0\;\left(i\neq r\right).$$
**Step** **III** **Computation of the cluster center **$V_{i}\left(k+1\right)$
  (8) $$V_{i}\left(k+1\right)=\frac{\sum_{j=1}^{n}u_{ij}^{m}\left(k\right)x_{j}}{\sum_{j=1}^{n}u_{ij}^{m}\left(k\right)}.$$
**Step** **IV** **Iteration or termination**
  - If $∥V_{i}\left(k+1\right)-V_{i}\left(k\right)∥<\epsilon$ or k>K, iteration terminates at this instant.
  - Otherwise, k=k+1 and back to **Step II**.

FCM increases the optimizing capability of HCM, but it decreases the convergence rate [47]. Choosing a higher value of *c* results in over-segmentation/clustering of the 3D point cloud. This ultimately helps segment small and very close objects as well.

**Figure 7:**
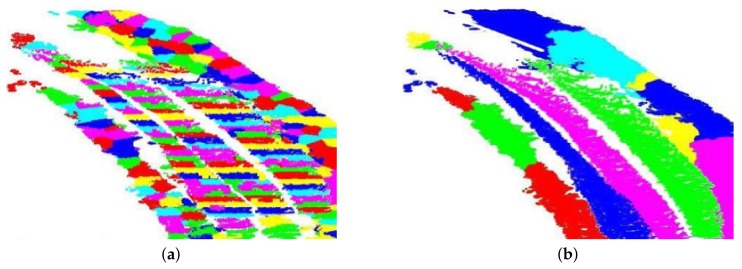
Segmentation results. (**a**) The over-segmented point cloud after fuzzy clustering, (**b**) the results after the agglomerative clustering step.

Once the point cloud is over-segmented into smaller segments, they are grouped together to form larger objects. The smaller segments are grouped together along the longer dimension of the ditch. The direction of the longer dimension is simply found by analyzing the bounding box of the 3D point cloud of the ditch. As we are searching for pipe-like structures, supposedly along the longer dimension of the ditch, subsequent colinear segments are grouped together to form larger segments as shown in Figure 7. These larger segments represent potential pipe-like objects.

### 3.4. Modeling of Detected Pipes

Once potential pipe-like objects are segmented in the point cloud, they are first analyzed to determine whether they belong to a cylindrical pipe and, if so, their parameters are estimated for modeling.

The 3D points belonging to a segmented object are first divided into a finite number of equal segments along the object’s length (i.e., $\;\frac{Segment\_Length}{l_{n}}$ where $l_{n}$ is taken as 25). This helps to better estimate cylindrical parameters for curved pipe segments. A cylinder model is then used with MSAC (M-estimator SAmpling Consensus) [48] to parameterize the potential pipe surface for each segment which best fits the sampled measurements. This is implemented with two nested MSAC subroutines: First is line fitting, and second is the circle fitting of the projection of points onto the plane orthogonal to the line.

In a set of points Λ, we choose randomly two points $\lambda_{a}$ and $\lambda_{b}$ such that $\lambda_{a}\neq \lambda_{b}$. A line u is fitted through $\lambda_{a}$ and $\lambda_{b}$ and all the points in Λ are then projected onto the plane orthogonal to *u* (i.e., $orth_{u}$) to obtain a set $V\in R^{2}$. The density of *V* is computed and a subset *W* is chosen to separate points of high density. This helps reduce the effect of different types of noise, for example due to branches, during the estimation/parameterization of the cylinder radius. In order to fit a circle to the points in *W*, a MSAC subroutine is then used. Three unique points $w_{c}$, $w_{d}$, and $w_{e}$ are chosen randomly as the minimal sample set (MSS) and a circle (defined by its center $c\in R^{2}$) is estimated/parameterized based on the Kasa Method [49]. Using Equation (9), error is computed for all points in *W* and, based on $∥\epsilon_{i}∥<\epsilon_{0}$, the inlier set *I* is determined. The MSAC model is relatively insensitive to the choice of $\epsilon_{0}$.

(9) $$\epsilon_{i}=\sqrt{{\left(w_{i}-c\right)}^{T}\left(w_{i}-c\right)}-r.$$

The outer/top-level MSAC cylinder fit subroutine continues until a stopping criterion is reached. It returns the cylinder parameters which instantiated *I* with the highest likelihood as the best model. The algorithm is summarized in Algorithm 1.

Once the cylindrical parameters (line and diameter) for each segment is estimated, the different segment lines are concatenated and second order polynomial estimation is used to obtain a smooth center line. The radius R of the curved cylinder pipe is considered as the mean of the radii of all the different segments.

Even though the length of the cylinder pipe is equal to the length of the curve line estimated using the 3D points, the smooth center line is then extrapolated up to the full length of the ditch (limited by the length and width of the ditch) as the pipe curvature and cross-sectional diameter usually remain constant right through its length. This helps to obtain the complete pipe in the ditch and also helps cater for missing pipe segments due to lack of 3D points, occlusions, etc.

**Algorithm 1:** A summary of the proposed pipe modeling method.

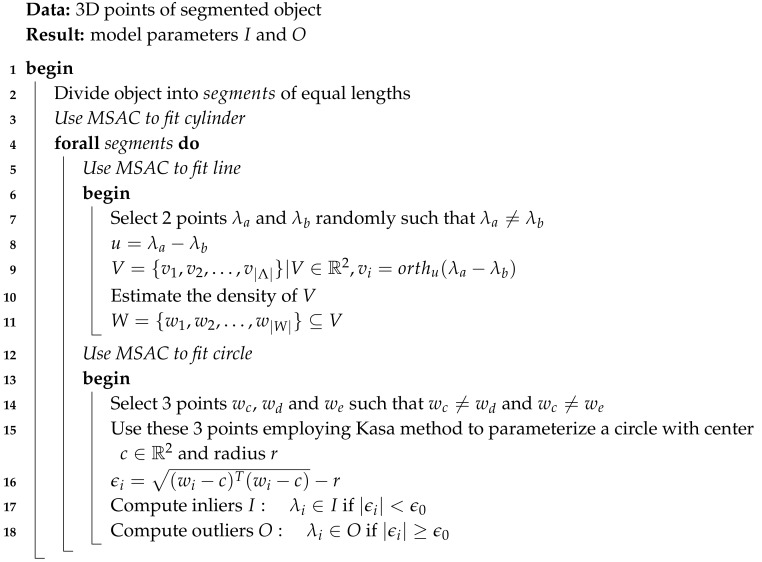



## 4. Experiments, Results, and Discussion

**Figure 8:**
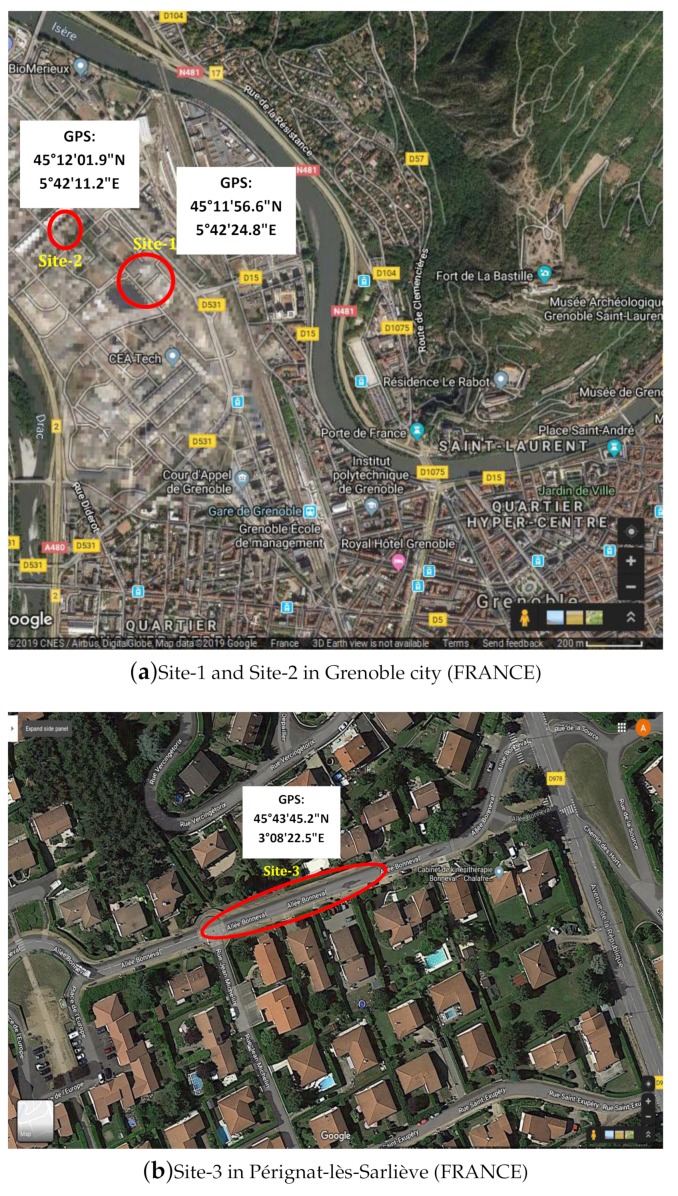
Location of sites shown in Google Maps (dated: 16 August 2019).

**Figure 9:**
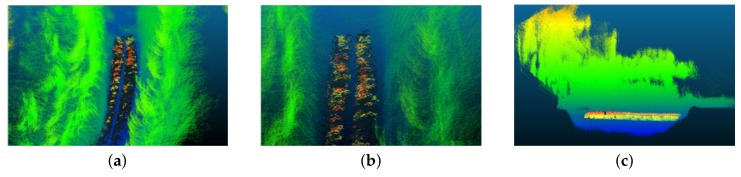

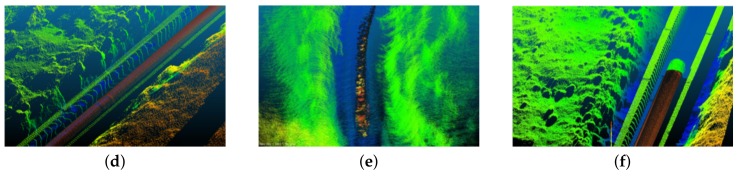
Some segmentation results from the three sites. Pipes are segmented in the 3D point clouds (**a**–**f**). The point clouds, apart from the segmented pipes, are colored based on the elevation of the 3D points with blue representing the lowest and red, the highest elevation.

**Figure 10:**
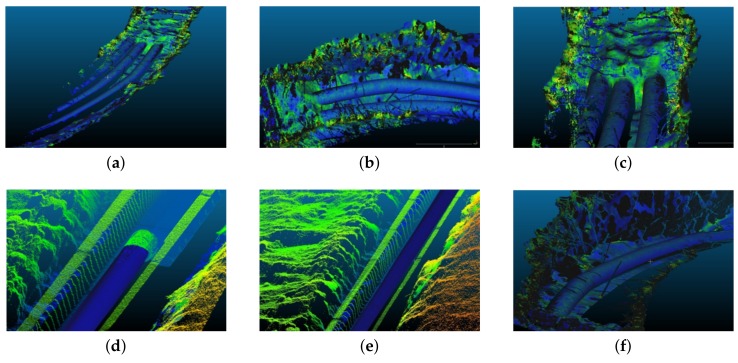
(**a**–**f**) Modeling results from the three sites. The modeled pipes are presented in blue in the 3D point clouds that are colored based on the elevation of the 3D points with blue representing the lowest and red, the highest elevation.

In order to evaluate the proposed method, real data were obtained using the portable LiDAR acquisition system explained in Section 3.1 at three different sites located in the city of Grenoble and Pérignat-lès-Sarlièves in France (location shown in Figure 8) at the time when underground pipes were being laid. The sites contained a number of ditches with different underground pipe networks. Qualitative results from some of these ditches are presented in Figure 9 and Figure 10. As seen in the figures, the pipes are generally well segmented and modeled by the proposed method.

**Figure 11:**
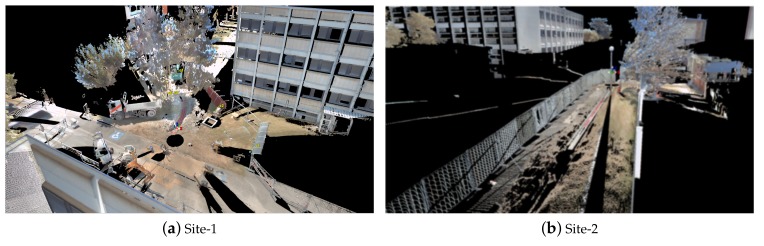

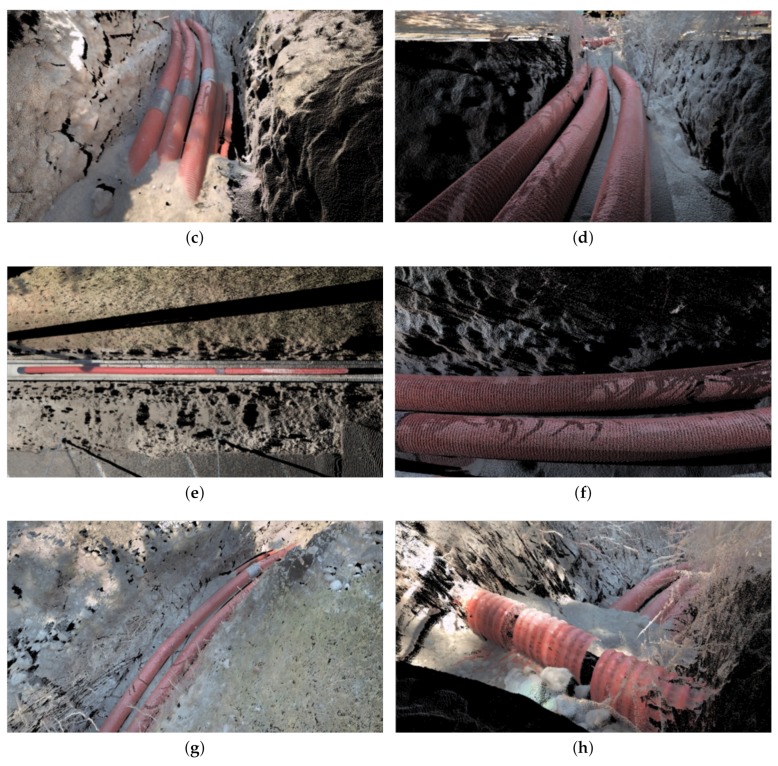
(**a**,**b**) The global 3D scan (ground truth) of Site-1 and Site-2 respectively, (**c**–**h**) shows the 3D scan (ground truth) of some of the different types of underground pipe networks found on the three sites.

For quantitative analyses ground truth was obtained, for each site, using a Leica P-20 total scan station [50]. The scan station consists of an accurate rotating 3D Laser scanner with a calibrated colored camera. The colored 3D scans were obtained from different positions and viewing angles around the ditch and then registered together to form a high resolution 3D colored point cloud [51] for each site as shown in Figure 11. The ground truth was obtained at a high accuracy of less than 5mm which is sufficient for evaluation purposes. With the help of the ground truth, different quantitative analysis were conducted as explained in the following sections.

### 4.1. Registration Accuracy

Some qualitative results for the registration process are presented in Figure 4 and Figure 5. For the quantitative results, as there is no standard method to measure the registration accuracy, we measured it in an indirect manner. In order to assess the registration accuracy, dimensions of different objects and features along the same *X*,*Y*, and *Z* axes in the registered 3D point cloud were analyzed and compared with those in the ground truth. The difference in the dimensions is considered primarily due to the registration errors. The different objects and features ‘*n*’ selected for the analysis mainly include large objects from the structured environment present around the ditch, and on the site, such as perpendicularity, length and height of nearby buildings and walls, dimensions of several windows, height and width of piping canals and lighting poles, etc. The dimensions of the same selected features from both the ground truth and the registered point cloud were then compared using the mean absolute error ratio (MAER) calculated for each site, along each of the three axes, as follows:

(10) $$MAER=\frac{1}{n}\sum_{i=1}^{n}\frac{∥{Dim}_{GT}^{i}-{Dim}_{M}^{i}∥}{{Dim}_{GT}^{i}}$$

where ${Dim}_{GT}^{i}$ and ${Dim}_{M}^{i}$ are the *i*-th dimension measured in the ground truth and the registered point cloud respectively. The results are presented in Table 1.

The higher value of MAER for the registered point cloud of Site-2 is due to the fact that there were not a lot of buildings or other built infrastructure (structured environment) around the ditch area and so as a result the registration process was somewhat hampered due to the rejection criterion explained in Section 3.1.

This shows the importance of having more structured environment around the ditch area to improve the registration quality.

### 4.2. Segmentation and Classification Results

The segmentation and classification results were evaluated at 3D point level. With the help of the available ground truth, the 3D points in the point cloud were manually labeled as belonging to pipes or otherwise. The results expressed in Table 2 are evaluated using different standard evaluation metrics as presented in [52]. Even though all these metrics are commonly used to evaluate such algorithms, the MCC (Matthews correlation coefficient) is insensitive to different class sizes (as is the case with our application, the number of pipe segment points are generally quite inferior as compared to points belonging to the surrounding environment, i.e., non-pipe points) and is considered as the most balanced measure.

Like other metrics, MCC is calculated using the values/counts of the true positives (i.e., correct detection of a 3D point belonging to a pipe), false positives, true negatives and false negatives. A coefficient value of +1 signifies a perfect prediction, 0 is no better than random prediction, and −1 implies total disagreement. The results, including an overall accuracy (ACC) and Matthews Correlation Coefficient (MCC) greater than 85% and +0.6, respectively, clearly show the efficiency and usefulness of the proposed method.

### 4.3. Modeling Accuracy

The segmented pipes are modeled as cylindrical tubes/pipes. Two main parameters of these modeled pipes are evaluated (i.e., the position of center line and the radius) and compared with the corresponding values obtained from the ground truth. The position of the center line was measured along the *X*, *Y*, and *Z* axes, using a common point as a measurement reference. The results are evaluated using the standard deviation σ as a measure to evaluate how much the center line of the modeled pipes deviates from the one in the ground truth, and Equation (10) is used to calculate error in the diameter estimation. The results are presented in Table 3.

**Figure 12:**
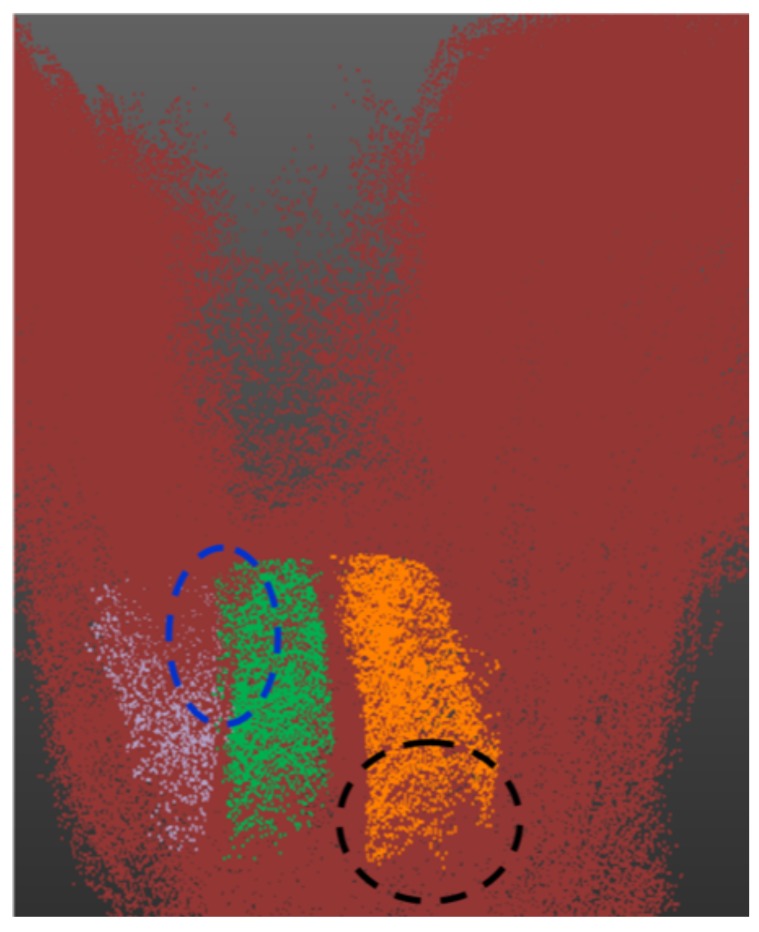
Black circle shows the missing 3D points from the bottom part of the segmented pipe due to occlusion and extraction as ground points whereas blue circle marks the incorrect segmentation with close objects due to jump edges.

**Figure 13:**
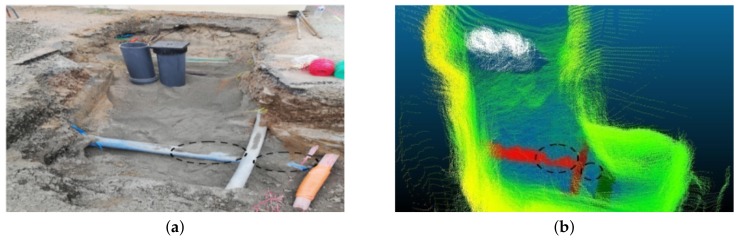
In (**a**) and (**b**), black circle marks the portion of the pipe incompletely segmented due to occlusions (covered by sand).

A higher standard deviation is observed in the case of the *Z*-axis due to a lack of 3D points belonging to the bottom part of the pipe, as it is usually occluded during scanning, and also some lower parts of the pipe were extracted out as part of ground points (see Figure 12 for example) and so as a result there is a larger deviation from the true center line along the *Z*-axis. It is for this reason that the dimensions along the *X*-*Y* axis are used to estimate the pipe diameter rather than the height axis *Z*. The error in the diameter measurement is strongly affected by the segmentation errors, especially due to jump edges between two adjacent objects in the unstructured environment as shown in Figure 12, and also due to occlusions as shown in Figure 13 and discussed in Section 3.3.2. In Figure 13, it can be clearly seen that the vertical pipe structures (white in Figure 13b) are not detected/segmented as they are not considered as pipes by the proposed method, and also due to sand covering parts of the pipe (marked by black circle marks).

## 5. Conclusions

In this paper, we present a portable, automated mapping solution for the 3D mapping and modeling of underground pipe networks during renovation and installation work, when the infrastructure is being laid down in open trenches. The system is used to scan the trench and the 3D scans obtained from the system are registered together to form a 3D point cloud of the trench containing the pipe network using a modified global ICP method. The pipes are segmented in the resulting 3D point cloud using fuzzy C-means clustering and then modeled using a nested MSAC algorithm. The proposed method is evaluated on real data from three different sites, containing several different types of pipes. An overall registration error of less than 7% is obtained, and it is observed that the proposed registration method gives higher registration accuracy (i.e., lower registration errors) in the presence of structured environment around the site, as compared to less or no structured environment. An overall segmentation accuracy and modeling error of 85% and less than 5% respectively, are also reported. These results are sometimes hampered due to occlusions and jump edges. In order to improve the segmentation and consequently the modeling results, 2D cameras could be added to the portable system in future works. The 2D colored images would be registered with the 3D scans to obtain a colored 3D point cloud. This extra information would provide additional discriminating factors enabling better segmentation [53].

The evaluated results demonstrate the efficacy and the technical prowess of the proposed solution. This solution is also an important step in reducing operational costs and improving mapping accuracy of underground pipe networks.

## Figures and Tables

**Table 1:** The mean absolute error ratio (MAER) along the *X*, *Y*, and *Z* axes for *n* number of reference dimensions chosen in the respective datasets.

	MAER			
	*X* Axis	*Y* Axis	*Z* Axis	$s=\sqrt{X^{2}+Y^{2}+Z^{2}}$
Site-1 (*n* = 30)	0.025	0.031	0.043	0.058
Site-2 (*n* = 15)	0.035	0.048	0.059	0.084
Site-3 (*n* = 25)	0.027	0.039	0.046	0.066
			**Average**	0.069 (=6.9%)

**Table 2:** The results evaluated using different standard evaluation metrics.

		Site-1	Site-2	Site-3
ACC	Accuracy	0.874	0.850	0.861
PPV	Positive Predictive Value	0.846	0.810	0.833
NPV	Negative Predictive Value	0.851	0.825	0.841
FDR	False Discovery Rate	0.154	0.190	0.167
$F_{1}$	$F_{1}$ measure	0.710	0.610	0.690
MCC	Matthews Correlation Coefficient	+0.631	+0.602	+0.629

**Table 3:** The evaluation of pipe modeling accuracy for the three sites.

	$\sigma_{X}$ (m)	$\sigma_{Y}$ (m)	$\sigma_{Z}$ (m)	Diameter (MAER)
Site-1	0.017	0.018	0.051	0.051
Site-2	0.016	0.021	0.045	0.043
Site-3	0.014	0.018	0.041	0.045
			**Average**	0.046 (=4.6%)
